# Potential roles of the RGMa-FAK-Ras pathway in hippocampal mossy fiber sprouting in the pentylenetetrazole kindling model

**DOI:** 10.3892/mmr.2014.2993

**Published:** 2014-11-21

**Authors:** MING-YU SONG, FA-FA TIAN, YU-ZHONG WANG, XIA HUANG, JIA-LING GUO, DONG-XUE DING

**Affiliations:** Department of Neurology, Xiangya Hospital, Central South University, Changsha, Hunan 410008, P.R. China

**Keywords:** RGMa, FAK (Tyr397), Ras, mossy fiber sprouting, temporal lobe epilepsy, hippocampus

## Abstract

Mossy fiber sprouting (MFS) is a unique feature of chronic epilepsy. However, the molecular signals underlying MFS are still unclear. The repulsive guidance molecule A (RGMa) appears to contribute to axon growth and axonal guidance, and may exert its biological effects by dephosphorylating focal adhesion kinase (FAK) at Tyr397, then regulating the activation of Ras. The objective of this study was to explore the expression patterns of RGMa, FAK (Tyr397) and Ras in epileptogenesis, and their correlation with MFS. The epileptic models were established by intraperitoneal pentylenetetrazole (PTZ) injection of Sprague-Dawley rats. At 3 days and at 1, 2, 4 and 6 weeks after the first PTZ injection, Timm staining was scored at different time points in the CA3 region of the hippocampus and dentate gyrus. The protein levels of RGMa, FAK (Tyr397) and Ras were analyzed at different time points in the CA3 region of the hippocampus using immunofluorescence, immunohistochemistry and western blot analysis. Compared with the control (saline-injected) group, the expression of RGMa in the CA3 area was significantly downregulated (P<0.05) from 3 days and still maintained the low expression at 6 weeks in the PTZ group. The expression of FAK (Tyr397) and Ras was upregulated (P<0.05) in the PTZ groups. The Timm score in the CA3 region was significantly higher than that in the control group at different time points and reached a peak at 4 weeks. In the CA3 region, no obvious distinction was observed at the different time points in the control group. To the best of our knowledge, these are the first results to indicate that the RGMa-FAK-Ras pathway may be involved in MFS and the development of temporal lobe epilepsy.

## Introduction

Epilepsy is the most prevalent chronic neurological disorder. Although the exact pathogenic mechanisms remain unclear, mossy fiber sprouting (MFS) is a pathological phenomenon observed in both animal models of temporal lobe epilepsy (TLE) and brain sections of epileptic patients (1–3). The majority of studies support the hypothesis that MSF contributes to increased seizure susceptibility by forming recurrent excitatory circuits. However, the mechanisms underlying these structural changes are not fully understood. The hippocampal mossy fibers are normally guided to the CA3 and form synapses with the pyramidal cells. However, in the epileptic hippocampus, the mossy fibers abnormally innervate into the inner molecular layer and/or stratum oriens of the CA3, establishing hyperexcitable recurrent circuits, which is called MFS (4). Based on recent findings (5–7), MFS may be regarded as a result of the disruption of molecular mechanisms underlying axonal growth and axonal guidance.

The repulsive guidance molecule (RGM) was originally known as a glycosylphosphatidylinositol (GPI)-anchored glycoprotein involved in guiding axons in the developing chick retina (8). Repulsive guidance molecule A (RGMa) is one of the three homologs of RGM, and is principally expressed in the central nervous system (9). In addition to regulating axonal guidance, RGMa also functions as a myelin-derived neurite outgrowth inhibitor *in vitro* and *in vivo* (10–11). These studies indicate the essential role of RGMa in the neural circuit formation.

The Ras superfamily GTPase proteins play essential roles in mediating neurite outgrowth and maintaining growth cone morphology by regulating cytoskeletal reorganization. Ras, one of the GTPase proteins that is abundantly distributed in neuronal axons and growth cones, promotes axonal extension during development (12–13). A previous study has shown that RGMa may exert its biological effects by dephosphorylating focal adhesion kinase (FAK) at Tyr397, then regulating the activation of Ras (14). However, the role of RGMa in epileptogenesis and MFS remains unclear, and the potential signaling pathway remains unexplored.

Considering the possible functions of RGMa in the adult brain, we hypothesized that RGMa may also participate in the plastic changes that occur during TLE development through the RGMa-FAK-Ras pathway. In this study, we investigated this hypothesis using the pentylenetetrazole (PTZ) kindling model, which has been widely adopted as a model of synaptic rearrangement and neuronal plasticity in the epileptic brain (15,16).

## Materials and methods

### Animals and the PTZ model

A total of 120 adult male Sprague-Dawley rats (Animal Experimental Centre, Central South University, China) weighing 180–220 g were equally divided into a control and a PTZ group, each containing five subgroups of 12 rats. The PTZ group received a dose of 30 mg/kg PTZ (Sigma, St. Louis, MO, USA) intraperitoneally once per day until the rats were kindled or sacrificed (rats in the PTZ group were not kindled within 2 weeks), while the control rats were injected with an equal dose of saline. Rats were considered kindled when seizure attacks (score ≥3) occurred after each PTZ injection for five consecutive days using Racine’s scale system (17). At time points of 3 days and 1, 2, 4 and 6 weeks after the first injection, the rats were sacrificed by immediate decapitation under deep anesthesia, with the exception of the rats used for immunohistochemical anaylsis, which were perfused first.

All animals were treated humanely and this study conformed to the Guide for the Care and Use of Laboratory Animals published by the National Institutes of Health (18). All animal use protocols were approved by the Animal Ethics Committee of Central South University (Changsha, China).

### Behavior monitoring

The rats were observed for the occurrence of PTZ-induced seizures for at least 2 h immediately following the PTZ injection, each day, until kindling or sacrifice occurred. The convulsive behavior was evaluated as previously described (17): 0, no behavioral changes; 1, facial movements, ear and whisker twitching; 2, myoclonic convulsions without rearing; 3, myoclonic convulsions with rearing; 4, clonic convulsion with loss of posture; 5, generalized tonic-clonic seizures.

### Timm staining

At different time points, the rats were deeply anesthetized with 10% chloral hydrate and perfused intracardially with 300 ml saline, followed by 200 ml 0.1 M phosphate buffer (pH 7.2–7.6) containing 0.4% sodium sulfide and 200 ml 4% paraformaldehyde at 4°C. The brains were removed, fixed in 4% paraformaldehyde for 24 h, then transferred to 0.1 M phosphate buffer containing 30% sucrose, and finally cut into 30-μm coronal sections.

The sections were stained in the dark for 90 min in a solution containing 60 ml 50% gum arabic, 10 ml 2 M citrate buffer, 30 ml 0.5 M hydroquinone and 0.5 ml 17% silver nitrate. After washing in water, the slides were restained with Nissl solution (Beyotime Co., Shanghai, China). Following this, the slides were routinely dehydrated, cleaned and mounted with gum.

### Immunohistochemistry

At different time points, the rats were deeply anesthetized with 10% chloral hydrate and perfused intracardially with 300 ml saline and 200 ml 4% paraformaldehyde in 0.1 M phosphate buffer at 4°C, and then decapitated. The brains were removed and placed in 4% paraformaldehyde overnight, then transferred into 0.1 M phosphate buffer containing 20% and 30% sucrose. Subsequently, serial sections were cut at a thickness of 20 μm for analysis. The tissue sections were subjected to conventional rewarming and heat-induced antigen retrieval in 10 mM sodium citrate buffer at boiling point for 24 min, with supplementation of cool sodium citrate buffer every 6 min. Peroxidase and lipids were eliminated by the admixture of 1% hydrogen and methanol at 4°C for 30 min. After rinsing in 0.01 M phosphate-buffered saline (PBS), the sections were blocked using a 5% goat serum reagent at room temperature for 2 h and incubated with anti-RGMa (rabbit anti-rat polyclonal antibody; 1:50; Santa Cruz Biotechnology, Inc., Santa Cruz, CA, USA) and anti-FAK Tyr397 (rabbit anti-rat polyclonal antibody; 1:50; Santa Cruz Biotechnology, Inc.) overnight at 4°C. Subsequently, immunohistochemistry was performed according to the kit protocol of the Biotin-Streptavidin Horseradish Peroxidase (HRP) Detection system, using normal goat serum for inhibition, biotin-labeled goat anti-rabbit immunoglobulin (Ig)G and streptavidin/HRP (Zhongshan Golden Bridge Biotechnology Co., Ltd., Zhongshan, China).

### Immunofluorescence

The rewarming and antigen recovery of brain tissue sections were performed as above. Each section was permeabilized with 1% Triton X-100 (Sigma) in Tris-buffered saline with Tween 20 (TBST). After blocking with 10% goat serum (Zhongshan Golden Bridge Biotechnology Co., Ltd.) at room temperature for 2 h, each sample was incubated with anti-RGMa and anti-FAK Tyr397 at 4°C overnight. After rinsing in 0.01 M PBS, the sections were incubated with goat anti-rabbit IgG (1:1,000; Invitrogen Life Technologies, Carlsbad, CA, USA) in the dark for 2 h at room temperature.

### Western blot analysis

At different time points, rats in the control and PTZ groups were deeply anesthetized with chloral hydrate and immediately decapitated for western blot analysis. Tissues were snap-frozen in liquid nitrogen, and proteins isolated from the hippocampus were extracted using RIPA lysate buffer (Beyotime Co.). All proteins were denatured (95°C, 10 min) and chilled on ice (5 min), then electrophoresis was performed (10% SDS-PAGE). The proteins were transferred onto 0.45- or 0.22-μm polyvinylidene difluoride membranes (Pall Corporation, Port Washington, NY, USA), washed three times with TBST, and then blocked with 5% skimmed milk in TBS (room temperature, 2 h). The membranes were incubated at 4°C overnight with the appropriate primary antibodies (anti-RGMa rabbit anti-rat polyclonal antibody, 1:800; anti-FAK Tyr397 rabbit anti-rat polyclonal antibody, 1:500; anti-Ras rabbit anti-rat polyclonal antibody, 1:500; anti-GAPDH rabbit anti-rat polyclonal antibody, 1:1,000). Unbound antibodies were washed, and the membranes were subsequently incubated with HRP-labeled goat anti-rabbit IgG secondary antibodies (1:2,000 dilutions; Beyotime Co.) for 1 h (room temperature). After washing (3×15 min), the immunoreactive bands were visualized by enhanced chemiluminescence (Bio-Rad imaging system; Bio-Rad, Hercules, CA, USA) and quantified using Image Lab software (Bio-Rad).

### Statistical analysis

Data are expressed as the mean ± standard deviation. Intergroup differences in Timm scores were compared using the Mann-Whitney U test, while intragroup differences were compared using the Kruskal-Wallis H test and then the Nemenyi test for pairwise comparison. Differences among multiple groups were assessed by a one-way analysis of variance, and differences between two groups were evaluated using the independent samples t-test. P<0.05 was considered to indicate a statistically significant difference. All statistical analyses were performed using the Statistical Package for the Social Sciences, version 17.0 (SPSS, Inc., Chicago, IL, USA).

## Results

### Behavioral outcomes

With the exception of three rats that died as a result of persistent generalized tonic-clonic seizure at 3 days or at 2 weeks, and one rat that was not kindled, the PTZ-treated rats developed seizure activity of different degrees after continuous PTZ injections for 21–28 days (an average of 23.6±2.0 days). The PTZ-induced seizure activity usually occurred 5–10 min after injection with a duration of 5–20 min. No epileptiform activity was observed behaviorally in the control rats.

### Severity of MFS in the CA3 region is correlated with the evolution of seizure behavior

**Figure 1:**
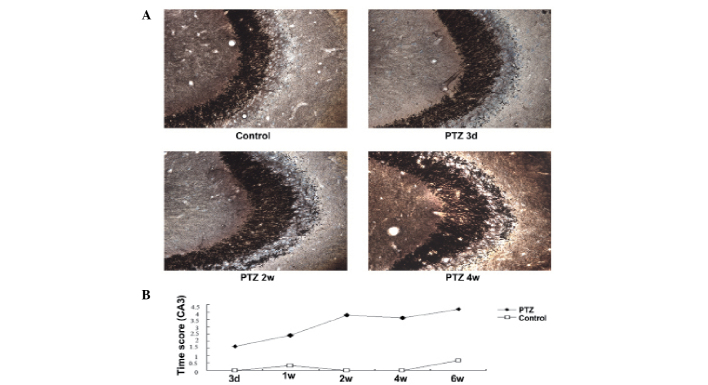
Timm staining in the CA3 region in the control and PTZ groups. (A) Timm granules in the control group and at different time points in the PTZ group in the CA3 region. (B) Time-dependent changes of Timm scores in the CA3 region in the PTZ and control group. PTZ, pentylenetetrazole.

The distribution of Timm granules in the stratum oriens of CA3 was rated on a scale of 0 to 5 according to published criteria (19). In control rats, there was no significant difference in Timm scores in the CA3 region (P>0.05). There were significant differences in the Timm scores in the CA3 region at each time point between the PTZ group and control group (P<0.05). The degrees of MFS in the CA3 were consistent with grades of seizure in the PTZ group and reached a peak at 4 weeks (Fig. 1). Conversely, Timm scores in the inner molecular layer were 0–1 throughout the experiment in the PTZ group, with no difference in the control group (data not shown).

### Expression of RGMa is significantly downregulated during PTZ kindling progression

**Figure 2:**
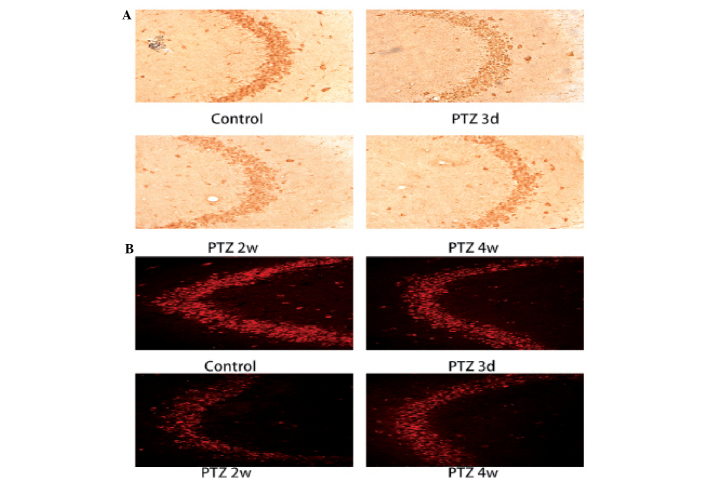
Expression of RGMa in the hippocampus by immunohistochemistry and immunofluorescence. (A) Immunohistochemistry staining in the CA3 region of the hippocampi in the control group and at different time points in the PTZ group. (B) Single immunofluorescence staining was performed on sections using an antibody specific to RGMa. RGMa, repulsive guidance molecule A; PTZ, pentylenetetrazole.

**Figure 3:**
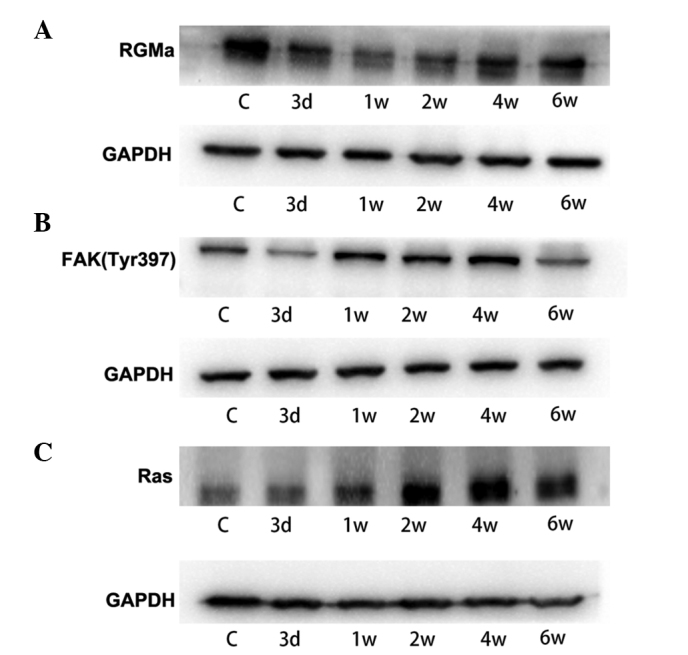
Expression of RGMa, FAK (Tyr397) and Ras by western blot analysis. (A) Western blot analysis showed the decreased expression of RGMa from 3 days, which was maintained at 6 weeks in the PTZ group. (B) The expression of FAK (Tyr397) increased markedly from 1 week and reached a peak at 4 weeks, then declined at 6 weeks. (C) Western blot analysis revealed increased expression of Ras from 1 week, which reached a peak at 4 weeks in the PTZ group. RGMa, repulsive guidance molecule A; FAK, focal adhesion kinase; PTZ, pentylenetetrazole.

RGMa expression was mainly observed in the neuronal membrane in the CA3 region of the hippocampus. Compared with the control group, the expression of RGMa in pyramidal cells of the CA3 region was significantly downregulated (P<0.05) in the PTZ group; it decreased markedly from 3 days and maintained the low expression at 6 weeks (Figs. 2 and 5A). No obvious distinction was observed in the CA3 region at different time points in the control group. The western blot analysis demonstrated similar results (Figs. 3A and 5C).

### Expression of FAK (Tyr397) and Ras is significantly upregulated during PTZ kindling progression

**Figure 4:**
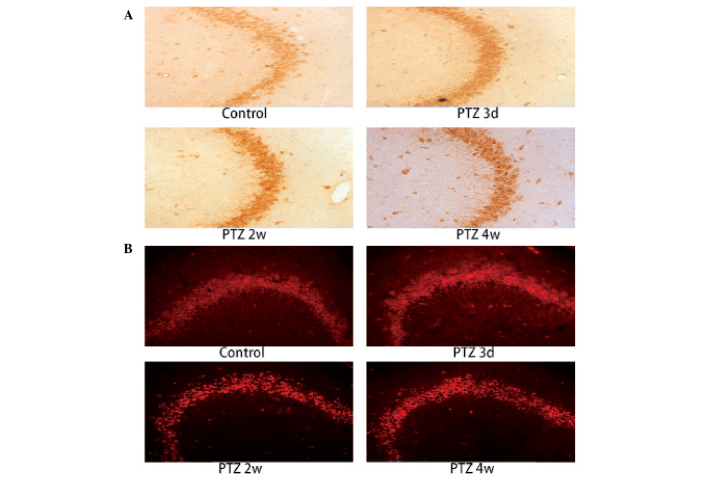
Expression of FAK (Tyr397) in the hippocampus by immunohistochemistry and immunofluorescence. (A) Immunohistochemistry staining in the CA3 region of the hippocampi in the control group and at different time points in the PTZ group. (B) Single immunofluorescence staining was performed on sections using an antibody specific to FAK (Tyr397). FAK, focal adhesion kinase; PTZ, pentylenetetrazole.

**Figure 5:**
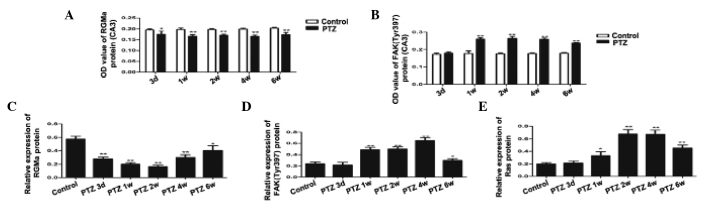
Expression of (A) RGMa and (B) FAK (Tyr397) in the CA3 area of the hippocampus, as assessed by immunohistochemical staining in the control and the PTZ groups. Compared with the control group, RGMa expression was significantly downregulated as early as 3 days post PTZ injection and the low expressionwas maintained at 6 weeks; FAK (Tyr397) was significantly increased in 1 week, reached a peak at 2 weeks and exhibited a decline at 6 weeks. Expression of (C) RGMa, (D) FAK (Tyr397) and (E) Ras in the hippocampus by western blot analysis. GAPDH was used as the loading control. Quantification of the western blot results revealed that RGMa expression decreased at all time-points in the PTZ group compared with the control and that the expression of FAK (Tyr397) increased in the PTZ compared with the control group. Ras was significantly increased after 1 week, reached a peak at 4 weeks and declined at 6 weeks.. The data are expressed as the mean ± SD. ^*^P<0.05, compared with the control; and ^**^P<0.01, compared with the control. RGMa, repulsive guidance molecule A; FAK, focal adhesion kinase; PTZ, pentylenetetrazole.

From the western blot analysis, the expression of FAK (Tyr397) and Ras were significantly upregulated (P<0.05) in the PTZ group. The expression of FAK (Tyr397) increased markedly from 1 week and reached a peak at 4 weeks, then declined at 6 weeks (Figs. 3B and 5D). The expression of Ras increased from 1 week and reached a peak at 4 weeks, then began to decline at 6 weeks (Figs. 3C and 5E). The immunohistochemistry and immunofluorescence results were in accordance with the western blotting (Figs. 4 and 5B). Figs. 4A and B show that the expression of FAK (Tyr397) accumulated mainly in the cytoplasm in the CA3 region. No obvious distinction was observed in the CA3 region at different time points in the control group.

## Discussion

This study examined the potential roles of RGMa and its potential downstream molecules on epileptogenesis and MFS using the PTZ kindling rat model. It was found that MFS preceded the appearance of spontaneous recurrent seizures (SRS) in the PTZ kindling model. The RGMa-FAK-Ras pathway was correlated with the progression of MFS.

With regard to the fact that MFS preceded the appearance of SRS, it is known that the occurrence and development of epilepsy are usually associated with neuronal loss (20), MFS (21) and synaptic reorganization in the hippocampus (22). The present study demonstrated that the degree of aberrant MFS was consistent with the severity of the seizure and that MFS can precede the occurrence of SRS. Although, there are also some reservations. Other studies have demonstrated that MFS is not associated with the progression of spontaneous seizures (23–24). However, in the current study, MSF was observed on day 3, which was before the appearance of SRS, indicating that MFS was more likely to be the cause of SRS.

The present study found that RGMa plays a potential role in neuronal reorganization in the kindling rat hippocampus. In the development of the neural circuit, specific axonal guidance and growth factors are essential for guiding the axon to the proper projection area. These factors can suppress the spontaneous or aberrant axonal growth to maintain the established neural connections and participate in the regulation of synaptic plasticity. Four major classes of axon guidance and growth molecules, the ephrins, netrins, slits and semaphorins, have been reported to play a role in synaptic reorganization in the adult brain and thereby promote epileptogenesis (5–7). In this way, the axonal growth and guidance-related signal molecules have major therapeutic significance for epilepsy.

RGMa is a novel type of GPI-anchored glycoprotein with no significant homology to any other known guidance and growth molecules. It is not only an axonal guidance molecule but also a novel axonal growth inhibitor. GPI-anchored molecules encompass a large group of proteins of great functional diversity. These molecules can act through contact attraction or repulsion to regulate axonal pathfinding and growth. RGMa plays a role in axonal morphogenesis by regulating neurite extension and branching (25). At present, the research on RGMa is more focused on spinal cord injury and cerebral vascular diseases. (10,26–28) These studies indicate the essential role of RGMa in axonal growth, pathfinding and synaptic plasticity. The correlation between RGMa and epileptogenesis and MFS remains unknown. One case report has demonstrated a deletion of the RGMa gene in a child with epilepsy and mental deficiency (29), and Brinks *et al* (30) reported that RGMa is involved in the layer-specific innervation of perforant fibers to the dentate gyrus. The principal finding of the present study was that RGMa was significantly downregulated in the hippocampus at time points consistent with the development of aberrant MFS in a model of PTZ-induced TLE. Therefore, our present findings provide new insights into the morphogenesis of mossy fibers: RGMa signaling negatively regulates the branching of mossy fibers. These results are consistent with the previous studies showing that RGMa inhibits neurite branching and outgrowth by binding to its receptor neogenin (10,31). However, the molecular mechanisms of RGMa in MFS remain to be explored.

The current study found that RGMa may participate in the plastic changes through the FAK-Ras pathway. Both the axon growth and guidance are eventually realized through induced cytoskeletal rearrangement. Studies have shown that RGMa induces cytoskeletal rearrangement by regulating the activation of Ras and RhoA (14,32). Both Ras and RhoA belong to the Ras superfamily GTPase proteins, which orchestrate actin filament assembly and disassembly by controlling actin polymerization, branching and depolymerization (33,34). RhoA has been demonstrated to be associated with epilepsy (35); however, to date, research into Ras has focused on survival, growth, proliferation and differentiation of cells (36–38). Studies have shown that Ras may regulate axonal extension and guidance during development (12–13). However, it is unknown whether Ras functions as a mediator of kindling-induced structural changes, and how RGMa regulates the activation of Ras.

A potential candidate to mediate the flow of information from the extracellular environment to the cytoskeleton, and thereby to control axonal development, is FAK. FAK expression is enriched in cell bodies and growth cones (39); several extracellular cues, such as axonal growth and guidance factors, have been shown to function upstream of FAK (40–41), suggesting that it might regulate the interactions between growing neurites and the extracellular matrix. It can interact with a complex molecular network via multiple phosphorylation sites. While the best characterized FAK phosphorylation event is the autophosphorylation at Tyr397, this phosphorylation event is the first and most important step in FAK activation. Autophosphorylation of FAK at Tyr397 leads to the formation of phosphotyrosine docking sites for several classes of signaling molecules and phosphorylation at additional tyrosine residues (42), suggesting that autophosphorylation of FAK at Tyr397 may act as an essential intracellular adaptor. A previous study has revealed that RGMa could inactivate Ras via FAK dephosphorylation to induce growth cone collapse (14). Therefore, RGMa may also participate in the plastic changes that occur during TLE development through the RGMa-FAK-Ras pathway.

In the present study, it was found that FAK (Tyr397) and Ras were significantly upregulated in the hippocampus at time points consistent with the development of aberrant MFS. Therefore, we hypothesize that following a decrease of RGMa expression in the hippocampus, the degree of FAK phosphorylation at Tyr397 increases then upregulates the expression of Ras, which ultimately facilitates the pathfinding and synaptic specificity of MFS via cytoskeletal rearrangement, providing a structural basis for enhanced excitation and epileptogenesis in the hippocampus.

In conclusion, this study demonstrated that MFS is not the outcome of SRS. The results of this study indicate, for the first time, that RGMa may be involved in MFS and synaptic reorganization through the RGMa-FAK-Ras pathway. Understanding the molecular mechanisms underlying MFS may lead to therapeutic interventions that protect the brain from recurrent spontaneous seizures.
